# Exploration of Chemical Diversity and Antitrypanosomal Activity of Some Red Sea-Derived Actinomycetes Using the OSMAC Approach Supported by LC-MS-Based Metabolomics and Molecular Modelling

**DOI:** 10.3390/antibiotics9090629

**Published:** 2020-09-22

**Authors:** Noha M. Gamaleldin, Walid Bakeer, Ahmed M. Sayed, Yara I. Shamikh, Ahmed O. El-Gendy, Hossam M. Hassan, Hannes Horn, Usama Ramadan Abdelmohsen, Wael N. Hozzein

**Affiliations:** 1 Department of Microbiology, Faculty of Pharmacy, The British University in Egypt (BUE), Cairo 11837, Egypt; noha.gamaleldin@bue.edu.eg; 2 Center for Drug Research and Development, Faculty of Pharmacy, The British University in Egypt (BUE), Cairo 11837, Egypt; 3 Department of Microbiology, Faculty of Pharmacy, Beni-Suef University, Beni-Suef 62514, Egypt; waleed.ismail@nub.edu.eg (W.B.); ahmed.elgendy@pharm.bsu.edu.eg (A.O.E.-G.); 4 Department of Pharmacognosy, Faculty of Pharmacy, Nahda University, Beni-Suef 62513, Egypt; Ahmed.Mohamed.Sayed@nub.edu.eg; 5 Department of Microbiology & Immunology, Faculty of Pharmacy, Nahda University, Beni-Suef 62513, Egypt; yara.shamikh@nub.edu.eg; 6 Virology Department, Egyptian Center for Research and Regenerative Medicine (ECRRM), Cairo 11517, Egypt; 7 Department of Pharmacognosy, Faculty of Pharmacy, Beni-Suef University, Beni-Suef 62514, Egypt; hossam.mokhtar@nub.edu.eg; 8 Independent Researcher, 69126 Heidelberg, Germany; hannesdhorn@gmail.com; 9 Department of Pharmacognosy, Faculty of Pharmacy, Minia University, Minia 61519, Egypt; 10 Department of Pharmacognosy, Faculty of Pharmacy, Deraya University, New Minia 61111, Egypt; 11 Bioproducts Research Chair, Zoology Department, College of Science, King Saud University, Riyadh 11451, Saudi Arabia; 12 Botany and Microbiology Department, Faculty of Science, Beni-Suef University, Beni-Suef 62512, Egypt

**Keywords:** actinomycetes, co-fermentation, *Micromonospora*, metabolomics, docking, antitrypanosomal, OSMAC

## Abstract

In the present study, we investigated the actinomycetes associated with the Red Sea-derived soft coral *Sarcophyton glaucum* in terms of biological and chemical diversity. Three strains were cultivated and identified to be members of genera *Micromonospora*, *Streptomyces*, and *Nocardiopsis*; out of them, *Micromonospora* sp. UR17 was putatively characterized as a new species. In order to explore the chemical diversity of these actinobacteria as far as possible, they were subjected to a series of fermentation experiments under altering conditions, that is, solid and liquid fermentation along with co-fermentation with a mycolic acid-containing strain, namely *Nocardia* sp. UR23. Each treatment was found to affect these actinomycetes differently in terms of biological activity (i.e., antitrypanosomal activity) and chemical profiles evidenced by LC-HRES-MS-based metabolomics and multivariate analysis. Thereafter, orthogonal projections to latent structures discriminant analysis (OPLS-DA) suggested a number of metabolites to be associated with the antitrypanosomal activity of the active extracts. The subsequent in silico screenings (neural networking-based and docking-based) further supported the OPLS-DA results and prioritized desferrioxamine B (**3**), bafilomycin D (**10**), and bafilomycin A1 (**11**) as possible antitrypanosomal agents. Our approach in this study can be applied as a primary step in the exploration of bioactive natural products, particularly those from actinomycetes.

## 1. Introduction

Actinomycetes are considered talented producers of bioactive secondary metabolites that are spread across a wide range of terrestrial and aquatic habitats. These Gram-positive bacteria can also establish symbiotic relationships with other higher organisms like plants, animals, and insects [1,2,3]. During the last 70 years, actinomycetes from terrestrial environments have been extensively investigated and have gained great success in providing much essential therapeutics (e.g., antibiotics and anticancer agents). On the other hand, these prolific producers of specialized metabolites are still poorly explored in marine environments [4,5,6]. Additionally, the vast majority of marine-derived bioactive natural products have been reported from marine actinomycetes [7,8]. Thus, further exploration of actinomycetes from the marine habitats, notably those associated with marine macroorganisms (e.g., sponges and soft coral), is linked to an increased discovery rate of new species with a new genetic makeup that can eventually become a source of new chemistry [9,10,11,12,13]. The one strain many compounds (OSMAC) approach has gained a lot of interest in recent years. Applying this strategy by altering the fermentation conditions of a given microorganism can expand its genetic expression, thus its potential as a producer of unusual specialized metabolites can be exploited [14,15,16,17,18]. Chemical and biological elicitations (i.e., co-cultivation) have been reported among the most successful methods used as an application of the OSMAC approach [19,20,21,22].

**Figure 1:**
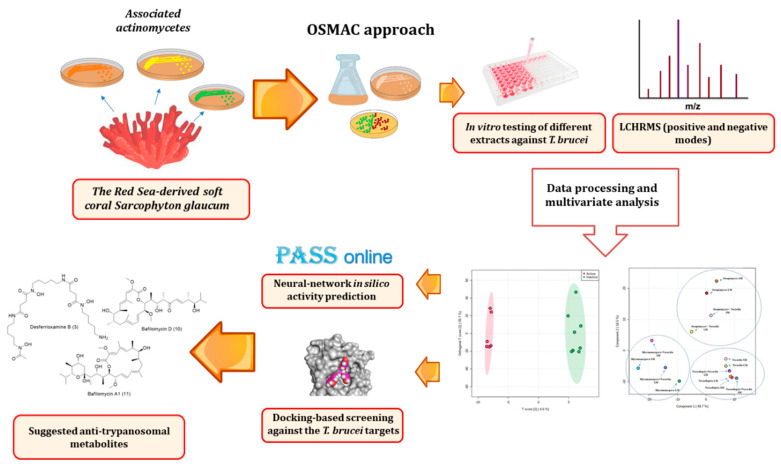
The applied approach in the present investigation. OSMAC, one strain many compounds; PASS, Prediction of Activity Spectra for Substances.

Trypanosomiasis is considered one of the fetal neglected tropical diseases and threatens more than 65 million people in sub-Saharan Africa [6], thus the enormous biosynthetic capacity of actinomycetes can provide potential drug candidates against this deadly parasite. Accordingly, we decided to extend our investigation of marine-derived actinobacteria, notably those associated with the Red Sea soft corals. Herein, we putatively identified a new member of the genus *Micromonospora* along with the other two actinomycetes species from the Red Sea-derived soft coral *Sarcophyton glaucum*. Additionally, we studied the effect of altering culturing conditions of these actinomycetes on their metabolic profiles using LC-HRES-MS-based metabolomics followed by multivariate analysis. We also screened the extracts derived from each culturing treatment for their antitrypanosomal activity, and subsequently, we suggested a number of potential metabolites to be associated with the antitrypanosomal activity of the active extracts using orthogonal projections to latent structures discriminant analysis (OPLS-DA) together with two different methods of virtual screenings (Prediction of Activity Spectra for Substances (PASS) prediction and docking). The strategy applied in the present study is depicted in Figure 1.

## 2. Materials and Methods

### 2.1. Sarcophyton Glaucum Collection

The soft coral material was collected from an area at the Red Sea that lies about 5 km to the north of Hurghada (latitudes 27°17′01.0” N and longitudes 33°46′21.0” E; and depth ranged from 3 to 6 m).

### 2.2. Chemicals

All chemicals and reagents used in the present study were of high analytical grade, purchased from Sigma Chemical Co Ltd. (St Louis, MO, USA).

### 2.3. Actinomycetes Isolation

Soft coral biomass was transferred to a plastic bag containing seawater and transported to the laboratory for further processing. Representative specimens were rinsed with sterile seawater, cut into smaller pieces of ca. 1 cm^3^, and subsequently thoroughly homogenized in a sterile mortar with 10 volumes of sterile seawater. The resulting supernatant was diluted in ten-fold series (10^−1^, 10^−2^, 10^−3^), and then streaked on agar plates using International *Streptomyces* Project-2 (ISP2) medium. All media were supplemented with 0.2 µm pore size filtered cycloheximide (100 µg/mL), nystatin (25 µg/mL), and nalidixic acid (25 µg/mL) to facilitate the isolation of slow-growing actinobacteria. Nystatin and cycloheximide were added to inhibit fungal growth, while nalidixic acid was added to inhibit many fast-growing Gram-negative bacteria. All media were supplemented with Difco Bacto agar (18 g/L) and prepared in 1 L artificial seawater (NaCl 234.7 g, MgCl_2_.6 H_2_O 106.4 g, Na_2_SO_4_ 39.2 g, CaCl_2_ 11.0 g, NaHCO_3_ 1.92 g, KCl 6.64 g, KBr 0.96 g, H_3_BO_3_ 0.26 g, SrCl_2_ 0,24 g, NaF 0.03 g, and ddH_2_O to 10.0 L). The inoculated Petri’s dishes were incubated at 30 °C for 6–8 weeks. Distinct colony morphotypes were picked and re-streaked until visually free of contaminants. Long-term storage of isolated strains was achieved in medium supplemented with 30% glycerol at −80 °C.

### 2.4. Molecular Identification

Molecular identification of the recovered actinomycetes was achieved by 16S rDNA sequencing according the previously reported methods using the universal primers 27F (5′-AGAGTTTGATCCTGGCTCAG3′) and 1492R (5′-GGTTACCTTGTTACGACTT-3′) [6,23,24]. There are many previous studies that describe the suitable similarity cut off for bacterial classification (from 99.5% to 97%). Some studies suggested that similarity <99.5% could be considered indicative of a new species within a known genus and others have found that 97% similarity was a suitable cutoff for identification at the species level. Hence, we preferred to use the strictest cutoff (<97%) to assign our isolated actinomycete as a new species [24]. The 16S rDNA sequences of each isolated actinomycete were analyzed using the Search and Classify option in the SINA web aligner. Using nucleotide Blast against nt and refseq_rna databases, the closest relatives and type strains were retrieved from GenBank. SINA web aligner v1.2.11 (variability profile: bacteria) was also used to calculate alignments. RAxML v8.2.12 (-f a -m GTRGAMMA) software was used to construct the maximum-likelihood tree with 100 bootstrap replicates. Afterward, this tree was visualized using interactive Tree Of Life (iTol) v5.5.

### 2.5. Fermentation and Extraction

Subsequently, the isolated actinomycetes (i.e., *Micromonospora* sp. UR17, *Nocardiopsis* sp. UR19, *Streptomyces* sp. UR23) were subjected to fermentation under a series of altering culturing conditions, that is, solid and liquid ISP2 media along with co-fermentation with mycolic acid-containing actinobacteria, *Nocardia* sp. UA 27 [6] also in solid and liquid ISP2 media. Solid media fermentations were performed in a number of Petri’s dishes (15 dishes for each treatment), while liquid fermentations were performed as follows: each strain was fermented in 2 L Erlenmeyer flasks each containing 1.5 L ISP2 medium. After incubation of monocultures and co-cultures, they were allowed to grow for 10 days at 30 °C while shaking at 150 rpm for liquid cultures. Thereafter, each actinomycete together with its fermentation medium was extracted by 100 mL of ethyl acetate three times, and then they were dried out and stored at 4 °C.

### 2.6. Metabolomic Profiling 

Ethyl acetate extract from each treatment was dissolved in methanol (1 mg/mL) for mass spectrometry analysis. Afterward, they were subjected to metabolomic analysis using LC-HR-ESI-MS according to our previously reported method [22]. Briefly, an Acquity Ultra Performance Liquid Chromatography system hyphenated with a Synapt G2 HDMS quadrupole time-of-flight hybrid mass spectrometer (Waters, Milford, Milford, MA, USA) was used for the LC-HRMS analysis. Both positive and negative ionization modes were applied together with a spray voltage of 4.5 kV. The capillary temperature was set at 320 °C, and mass range was set at m/z 150–1500. Afterward, the returned MS dataset was processed and extracted using MZmine 2.20 based on the established parameter. The processed data set was next subjected to molecular formula prediction and peak identification. The positive and negative ionization mode data sets from the respective extract were dereplicated using the DNP (Dictionary of Natural Products) database.

### 2.7. In Vitro Antitrypanosomal Activity 

The antitrypanosomal activity was tested following the protocol of Huber and Koella [6,25]. Briefly, 104 trypanosomes per ml of *Trypanosoma brucei brucei* strain TC 221 were added to complete Baltz medium. Trypanosomes were tested in 96-well plate chambers using various concentrations of test extracts at 10–200 µg/mL in 1% DMSO to a final volume of 200 µL. Then, 1% DMSO was used as control along with parasites without any test extracts and was applied simultaneously in each plate to show no effect of 1% DMSO. The plates were then incubated at 37 °C in an atmosphere of 5% CO_2_ for 24 h. Then, 20 µL of Alamar Blue was added and the activity was measured after 48 and 72 h by light absorption using an MR 700 Microplate Reader at a wavelength of 550 nm with a reference wavelength of 650 nm. The IC_50_ values of the test extracts were quantified by linear interpolation of three independent measurements. Suramin was used as a positive control (IC_50_ 0.23 μg/mL).

### 2.8. Statistical and Multivariate Analysis

LC-HRESMS-derived data were subjected to multivariate analysis (MVA) using MetaboAnalyst software [26]. Partial least squares discriminant analysis (PLS-DA) and orthogonal projections to latent structures discriminant analysis (OPLS-DA) were done to determine the variations in the metabolite composition in the samples and to highlight the metabolites that were probably linked to the observed antitrypanosomal activity of the tested extracts. The signal intensity of all variables was log10 transformed.

### 2.9. In Silico Biological Activity Predictions

The neural network-based software Prediction of Activity Spectra for Substances (PASS) [27] (www.way2drug.com) was used for further prioritization of the antitrypanosomal activity of the suggested compounds. This software is able to predict >4000 types of pharmacological and toxicological activities including their mechanism of action, with approximately 85% as acceptable precision, depending on the submitted compound structures that were subsequently screened utilizing the structure–activity relationship database (SARBase). The prediction results were expressed as probability scores (probably active “Pa” or probably inactive “Pi”). These calculated probability scores were determined by linking the structure and functional groups features in the tested molecules that matched or mismatched the specific activities listed in the software-associated database. The higher the Pa values, the more likely it was for the compound to display the suggested pharmacological activity on a scale of 0–1. Pa values higher than 0.5 mean a high experimental chance of the suggested pharmacological activity.

### 2.10. Docking Experiments

Molecular docking was carried out using Autodock Vina software [28]. The reported *T. brucei* target proteins (10 proteins, Table S1) were downloaded from the protein databank website (https://www.rcsb.org/), and then prepared using Autodock tools software [28]. The binding sites of the co-crystalized ligands were selected for the docking experiments, and the coordinates of these binding sites are listed in Table S1 in the Supplementary Material. Top-scoring binding poses with Root Mean square Deviation (RMSD) values less than 2 Å were then selected and visualized using Pymol software [29,30].

### 2.11. Statistical Analysis

All results in the present study were obtained from experiments performed in triplicates. The results were expressed as the means ± SEM of the indicated number of experiments (n ≥ 3). The statistical significance of differences between means was established by analysis of variance (ANOVA) with Duncan’s post hoc tests. *p*-values < 0.05 were considered to indicate statistical significance.

## 3. Results and Discussion

### 3.1. Identification and Phylogenetic Analysis of the Isolated Actinomycetes

**Figure 2:**
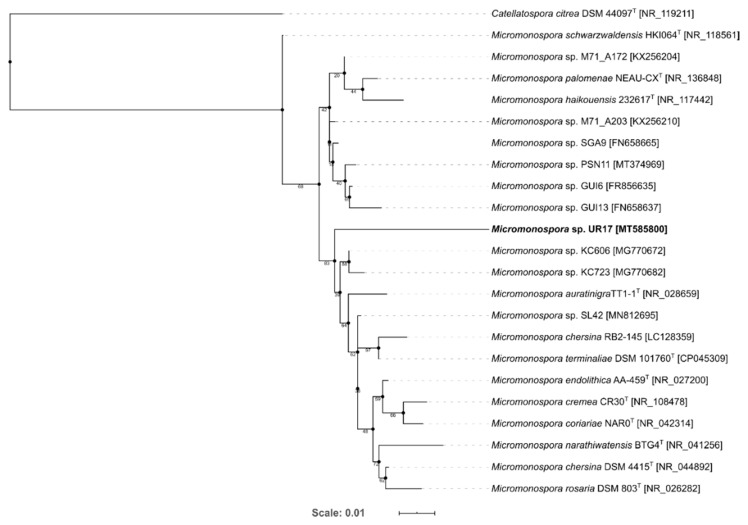
Maximum-likelihood tree of 23 *Micromonospora* representatives and 1 *Catellatospora* strain as an outgroup. Bootstrap values (100 resamples) are given in percent at the nodes of the tree. The isolate *Micromonospora* sp. UR17 obtained in this study is presented in bold.

In order to characterize the three actinomycetes that were recovered from the marine soft coral *Sarcophyton glaucum*, their 16S rRNA genes were sequenced and subsequently blasted against the database of NCBI GenBank. The results revealed that the three strains belonged to three different genera; that is, *Streptomyces*, *Nocardiopsis*, and *Micromonospora*, of which the *Micromonospora*-related strain was suggested to be a new species according to its 16S rRNA sequence similarity, which was lower than 97% (Table 1) [31]. The generated phylogenetic tree for *Micoromonospora* sp. UR17 reveals *Micromonospora* sp. KC606 to be the closest to *Micromonospora* sp. UR17, but did not show a specific cluster (Figure 2). *Micromonospora* is a well-known talented actinomycete and was considered as a huge pipeline of diverse, interesting bioactive specialized metabolites during the last 60 years; it is still considered as an untapped source for further novel ones [1]. Hence, this new strain of *Micromonospora* can be considered a promising source of bioactive chemical entities. However, simple fermentation procedures do not guarantee that the microorganism will express its secondary metabolites complete capacity. Accordingly, we conducted a series of fermentation processes under different conditions on this new strain along with the other two to maximize their specialized metabolites potential; in turn, the success rate of finding bioactive metabolites will increase.

### 3.2. In Vitro Antitrypanosomal Activity

All isolated actinomycetes were screened for their in vitro antitrypanosomal activity using the pathogenic strain *Trypanosoma brucei*. The extracts derived from *Streptomyces* sp. UR23 and *Micromonospora* sp. UR 17 upon culturing in solid medium or with the mycolic acid-containing strain *Nocardia* sp. UR27 showed interesting inhibitory activity towards *T. brucei*, with IC_50_ ranging from 2.4 to 16.6 µg/mL (Table 2). These findings highlight that both strains have the potential of producing bioactive antitrypanosomal metabolites only in certain conditions. Hence, all of these extracts were subsequently subjected to LC-HRES-MS-based metabolomic profiling to investigate the resulting change in the metabolites pattern in each actinomycete upon applying different culturing treatments.

### 3.3. Metabolomic Profiling and Multivariate Analysis

**Figure 3:**
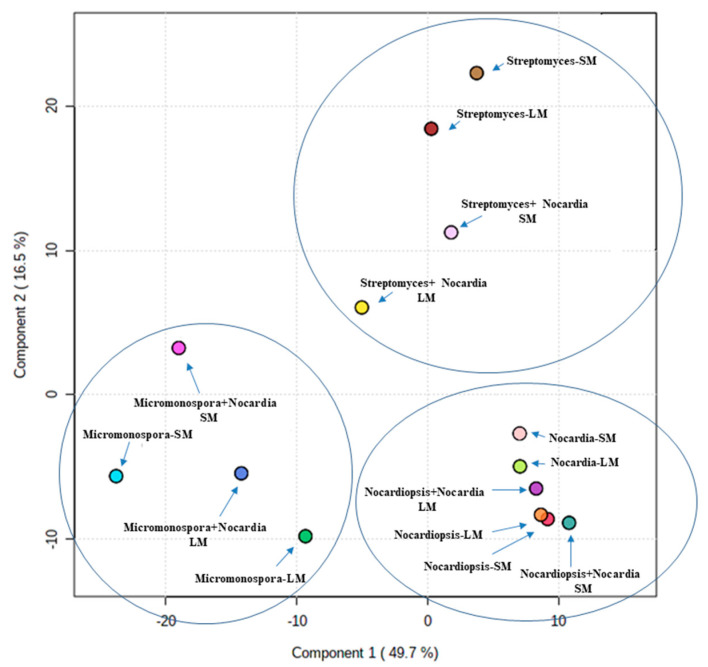
Partial least squares discriminant analysis (PLS-DA) scores plot of extracts derived from the three actinomycetes strains under different culturing conditions (*R*^2^ = 0.91, *Q*^2^ = 0.88). SM: solid media, LM: liquid media.

**Figure 4:**
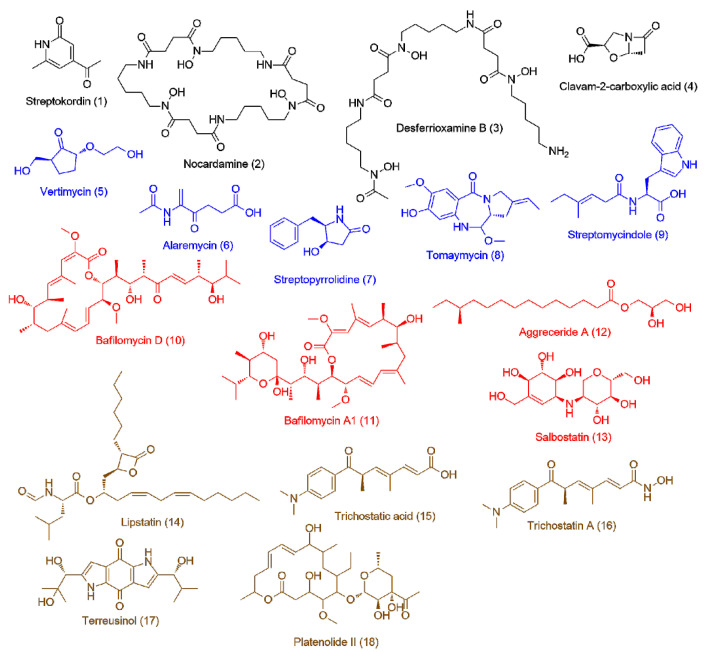
Induced metabolites (**1–18**) of *Streptomyces* sp. UR23-derived extracts: upon fermentation on the solid medium (black color, **1–4**), on the liquid medium (blue color, **5–9**), upon the co-fermentation on the solid medium (red color, **10–13**), and upon the co-fermentation in the liquid medium (brown color, **14–18**).

**Figure 5:**
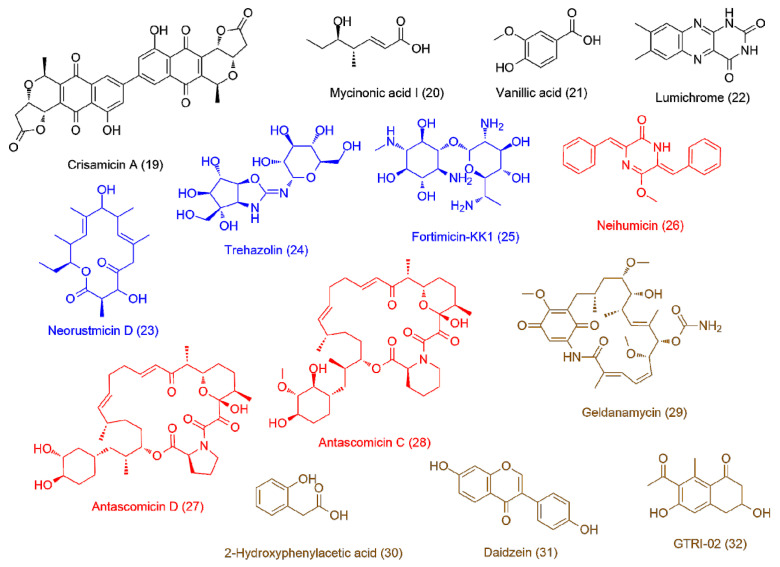
Induced metabolites (**19–32**) of *Micromonospora* sp. UR17-derived extracts: upon fermentation on the solid medium (black color, **19–22**), on the liquid medium (blue color, **23–25**), upon the co-fermentation on the solid medium (red color, **26–28**), and upon the co-fermentation in the liquid medium (brown color, **29–32**).

**Figure 6:**
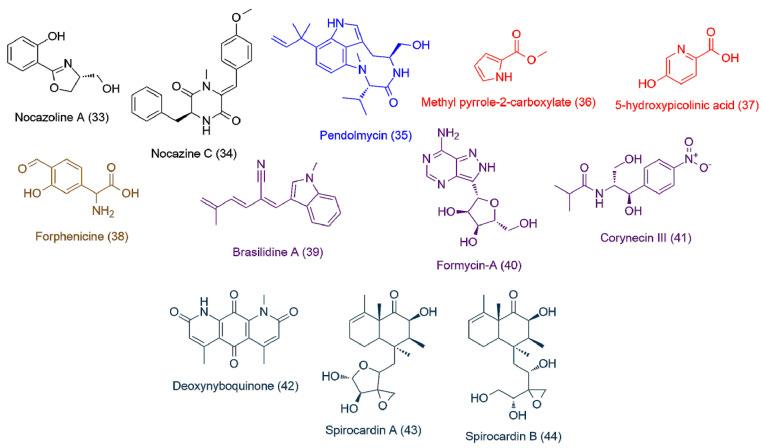
Induced metabolites (**33–44**) of *Nocardiopsis* sp. UR17-derived extracts: upon fermentation on the solid medium (black color, **33, 34**), on the liquid medium fermentation (blue color, **35**), upon the co-fermentation on the solid medium (red color, **36, 37**), upon the co-fermentation in the liquid medium (brown color, **38**), induced metabolites of *Nocardia* sp. UR27 on the solid medium fermentation (purple color, **39–41**), and induced metabolites of *Nocardia* sp. UR27 in the liquid medium fermentation (green color, **42–44**).

The LC-HRES-MS analysis resulted in tracing a total of 9784 peaks in the 14 extracts using positive and negative ionization modes. To observe the chemical variation between each actinomycete strain upon different culturing conditions, a PLS-DA model was generated from the resulted HRESIMS data. The PLS-DA scores plot (Figure 3, *Q*^2^ = 0.88, *R*^2^ = 0.91, the model was further validated by a permutation test of 1000 permutations, *p* < 0.001) showed a significant variation in the chemical profiles of the studied extracts, where the three actinomycete strains under the set of different treatments clustered separately from each other. Regarding the effect of different treatments on each strain, *Nocardiopsis* sp. UR19 showed no significant difference in its chemical profiles, where its extracts obtained from different treatments were closely clustered together (Figure 3). On the other hand, *Micromonospora* sp. UR17 and *Streptomyces* sp. UR23-derived extracts clustered more separately, indicating that these two strains responded in different ways upon each treatment in terms of metabolites production. PLS-DA-derived variable importance in projection (VIP) scores (Table 2) were used to investigate the induced metabolites upon each treatment (variables with VIP values >1.5). Table 2 and Figure 4, Figure 5 and Figure 6 summarize the dereplicated characteristic metabolites (i.e., induced) of the three actinomycete strains under each treatment. A taxonomic filter was applied during the dereplication process to select only hits that were related to the studied actinomycetes strains.

### 3.4. Bioactivity–Metabolites Correlation

**Figure 7:**
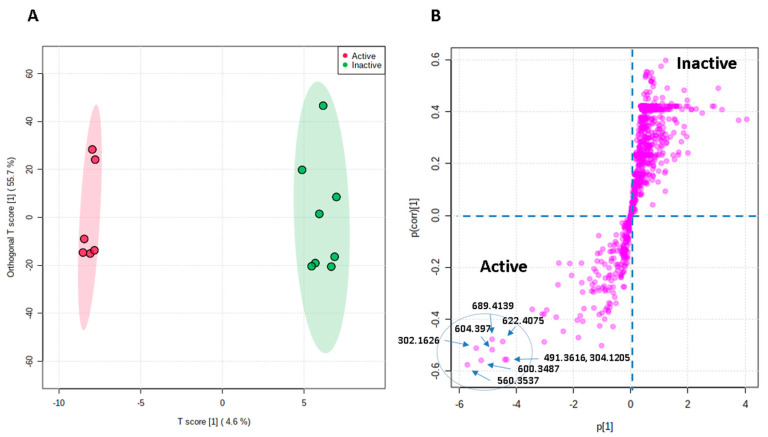
Orthogonal projections to latent structures discriminant analysis (OPLS-DA) score plot of active versus inactive extracts (**A**), together with its S-plot showing the highly correlated putatively active metabolites (**B**).

OPLS-DA was applied to investigate the possible metabolites that could be linked to the observed antitrypanosomal activity of the *Streptomyces* sp. and *Micromonospora* sp. UR17-derived extracts (i.e., obtained from solid fermentation and co-fermentation with *Nocardia* sp. UR27, Table 2). The resulting model revealed very good performance (goodness of models, *R*^2^ = 0.86) and prediction (predictive power of models, *Q*^2^ = 0.91). Moreover, cross-validation of the generated model was performed using a permutation test (1000 permutations), which indicated that none of the permutation-resulted models (i.e., based on random data) were better than the original one (*p* < 0.001), and thus our OPLS-DA model was unlikely overfitted. Extracts with IC_50_ values ≤20 µg/mL were considered active as antitrypanosomal, and the higher values were considered inactive. Figure 7A indicated a clear separation between active and inactive extracts, where each group was clustered together, suggesting the presence of common metabolites in these active extracts that could be correlated to their observed antitrypanosomal activity. Hence, the OPLS-DA-derived S-plots (Figure 7B) were used to pinpoint these bioactive discriminating metabolites in the active extracts. Table 3 illustrates the most influential metabolites (*p*-value ≤ 0.01) in the active extracts. Desferrioxamine B (**3**) (accurate mass = 560.3537) was putatively identified as the most important metabolite (*p* = 2.42 × 10^−6^) that may be linked to the antitrypanosomal activity of the active extracts, followed by nocardamine (**2**) (accurate mass = 600.3487, *p* = 3.33 × 10^−5^). Both metabolites are common *Streptomyces*-derived siderophores (i.e., iron-chelating metabolites) [36].

The third most important metabolite in the antitrypanosomal-active extracts was trichostatin A (**16**) (accurate mass = 302.1626, *p* = 1.22 × 10^−5^), which has been previously reported from several *Streptomyces* sp. [37,38], and has shown wide pharmacological effects (e.g., antifungal, anticancer, histone deacetylase, and tyrosinase inhibitor) [39].

The fourth most important metabolite was identified as bafilomycin D (**10**) (accurate mass = 604.397, *p* = 5.43 × 10^−4^), which has been reported from a number of actinomycetes and has been associated with a potent insecticidal activity [40].

The next top metabolite linked to the antitrypanosomal activity was antascomicin C (**28**) (accurate mass = 689.4139, *p* = 2.66 × 10^−4^), an antibiotic previously reported from several *Micromonospora* strains [1,41].

Bafilomycin A1 (**11**) (accurate mass = 622.4075, *p* = 1.12 × 10^−4^), which was also isolated from several actinomycetes species, particularly *Streptomyces*, has been identified as a potent and specific vacuolar-type H^+^-ATPase inhibitor, and thus has shown a wide range of antimicrobial and antiparasitic activities [42].

The next most discriminating metabolite in the antitrypanosomal group of extracts was identified as lipstatin (**14**) (accurate mass = 491.3616, *p* = 6.89 × 10^−3^). This metabolite was first isolated from *S. toxytricini*, and has been revealed as having very potent pancreatic lipase inhibition activity [43]. Interestingly, the saturated derivative of lipstatin (**14**), which is known as orlistat (**45**), has revealed significant antitrypanosomal activity [44].

The final most discriminating metabolite was identified as *Micromonospora* sp. UR17-derived metabolite neihumicin (**26**) (accurate mass = 304.1205, *p* = 3.11 × 10^−3^). This metabolite has been associated with interesting cytotoxicity against a wide range of cancer cell lines [1,45].

### 3.5. In Silico Predictions

**Figure 8:**
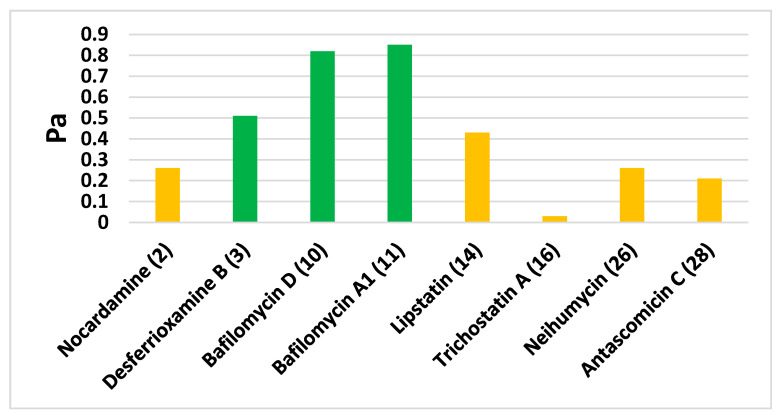
PASS prediction scores of metabolites suggested by OPLS-DA as possible antitrypanosomal agents. Pa scores > 0.5 indicated high possible antitrypanosomal experimental activity (green columns), while Pa < 0.5 indicated low possible antitrypanosomal experimental activity (orange color).

In order to support the MVA prediction of the antitrypanosomal activity-linked metabolites, we further subjected these selected metabolites (Table 3) to a neural network-based software called Prediction of Activity Spectra for Substances (PASS). The search algorithm of such activity prediction software depends on the structural similarity of a huge number of inhibitors reported for a wide range of biological targets. As shown in Figure 8, desferrioxamine B (**3**), bafilomycin D (**10**), and bafilomycin A1 (**11**) were found to be the most likely compounds to be associated with antitrypanosomal activity, with Pa scores of 0.51, 0.82, and 0.85, respectively.

**Figure 9:**
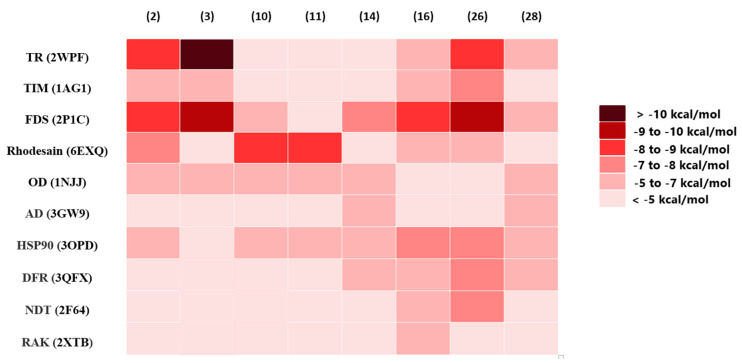
Heat map representing the binding energy scores resulting from the docking of OPLS-DA-derived metabolites against the known *T. brucei* molecular targets (10 proteins). Compounds achieving scores <−8 kcal/mol were categorized as top-scoring hits.

**Figure 10:**
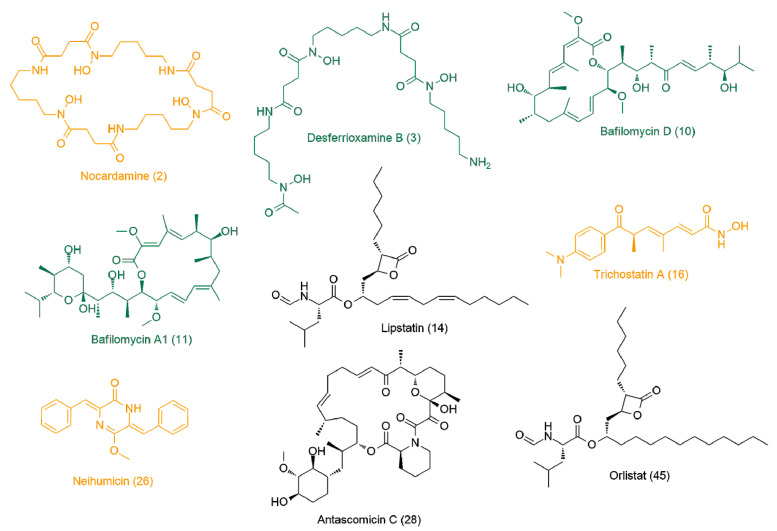
The most likely metabolites correlating with the observed antitrypanosomal activity of the active group of extracts according to OPLS-DA. Green color: metabolites (**3, 10, 11**) that showed high scores by PASS-based and docking-based virtual screenings; orange color: metabolites (**2, 16, 26**) that showed high scores by docking-base screening only; black color: metabolites (**14, 28, 45**) that did not show high scores upon any method of virtual screenings.

For further assessment, the most likely active metabolites (Table 3) were docked against 10 different molecular targets from *T. brucei* (i.e., inverse virtual screening). The docking scores are provided in Table S2. The top-scoring compounds were determined depending on their docking scores and binding modes in comparison with the reported co-crystallized ligands (binding energy < −8 kcal/mol). As summarized in Figure 9 and Figure 10, both trypanothione reductase (TR) and farnesyl diphosphate synthase (FDS) were found to be the most likely targets for nocardamine (**2**) and desferrioxamine B (**3**) and neihumicin (**26**), which achieved binding energy scores higher than the co-crystalized ligands (from −8.6 to −10.6 kcal/mol), while trichostatin A (**16**) achieved the highest binding score (−8.8 kcal/mol) against FDS only.

Regarding bafilomycin D (**10**) and bafilomycin A1 (**11**), their highest binding scores were recorded against rhodesain, with binding energies of −8.7 and −8.9 kcal/mol, respectively. Neither lipstatin (**14**) nor antascomicin C (**28**) was able to achieve a strong binding affinity toward any of the screened *T. brucei*. All top-scoring hits (Figure 9 and Figure 10) exhibited binding modes comparable with those of the native co-crystallized inhibitors. Computer-aided drug design together with neural networks deep learning-dependent processing has gained wide acceptance as an integral part of the drug discovery process, notably, prediction software that can reduce the required time and efforts for the screening of massive libraries of chemical compounds to figure out possible drug candidates. Such in silico techniques could be applied in drug discovery from natural sources, where they can prioritize a set of probably active hits among a complex mixture of metabolites present in a given natural crude extract; hence, the efforts required for isolation and characterization will be directed only to the top-scoring candidates.

## 4. Conclusions

Herein, we explored the diversity and the antitrypanosomal activity of the actinomycetes associated with the Red Sea-derived soft coral *S. glaucum*. Among the recovered actinomycetes, *Micromonospora* sp. UR 17 was putatively characterized as a new species according to its 16S rRNA sequence similarity in comparison with those reported in GenBank. Fermentation of these recovered actinomycetes under different conditions (OSMAC approach) was able to diversify their metabolites production, as evidenced by the LC-HRES-MS metabolomic profiling. Additionally, the biological activities of extracts derived from these actinomycetes upon each culturing treatment were completely different from each other; thus, such an observation also reflected the varying chemical composition of each extract. After subjecting these outcomes (LC-HRES-MS data and biological activity results) to MVA (PLS-DA and OPLS-DA), it was possible to highlight the biomarkers in each extract and to suggest a number of metabolites that can be linked to the observed antitrypanosomal activities. Virtual screenings through neural-networking and docking provided further support to the OPLS-DA-derived results and highlighted the most likely targets associated with the antitrypanosomal activity. Desferrioxamine B (**3**), bafilomycin D (**10**), and bafilomycin A1 (**11**) were classified as the most likely antitrypanosomal metabolites, as they were selected as top-scoring hits by both PASS-based and docking-based virtual screenings, among other OPLS-DA-derived hits. Our strategy in the present investigation could be considered as a preliminary evaluation step during the exploration of bioactive natural products from their corresponding natural sources, and in turn can save efforts of isolation for certain metabolites with a high potential for biological activity.

## Figures and Tables

**Table 1:** List of validly published strains of genus *Micromonospora* similarity calculated against strain *Micromonospora* sp. UR17 (Accession Number: MT585800).

Isolate	Accession ID	Identity [%]	Source	Ref
*Micromonospora terminaliae* DSM 101760	CP045309.1	93.28	Surface sterilized stem of Thai medicinal plant *Terminalia mucronata*	[32]
*Micromonospora cremea* CR30	NR_108478.1	93.00	rhizosphere of *Pisum sativum*	[33]
*Micromonospora palomenae* NEAU-CX1	NR_136848.1	92.81	Nymphs of stinkbug (*Palomena viridissima* Poda)	[34]
*Micromonospora rosaria* DSM 803	NR_026282.1	92.75	unknown	[35]

**Table 2:** Dereplication table of the induced metabolites determined for each actinomycete strain upon different culturing conditions and the antitrypanosomal activity of each resulting extract.

Culture Condition (IC_50_) *	No.	Retention Time	Mass	Molecular Formula	Dereplication	VIP ** Score
*Streptomyces* sp. UR23 on solid media (**16.6 ± 1.8**)	1	2.81	151.0623	C_8_H_9_NO_2_	Streptokordin	2.35
	2	3.44	600.3487	C_27_H_48_N_6_O_9_	Nocardamine	2.22
	3	8.83	560.3537	C_25_H_48_N_6_O_8_	Desferrioxamine B	2.15
	4	1.81	157.0368	C_6_H_7_NO_4_	Clavam-2-carboxylic acid	1.86
*Streptomyces* sp. UR23 in liquid media (>100)	5	2.76	174.0881	C_8_H_14_O_4_	Vertimycin	2.2
	6	2.06	185.0681	C_8_H_11_NO_4_	Alaremycin	1.98
	7	3.72	191.0947	C_11_H_13_NO_2_	Streptopyrrolidine	1.91
	8	4.1	304.1408	C_16_H_20_N_2_O_4_	Tomaymycin	1.85
	9	3.21	314.1629	C_18_H_22_N_2_O_3_	Streptomycindole	1.82
*Streptomyces* sp. UR23 with *Nocardia* sp. UR27 on solid media (**4.6 ± 1.5**)	10	7.81	604.397	C_35_H_56_O_8_	Bafilomycin D	1.97
	11	7.89	622.4075	C_35_H_58_O_9_	Bafilomycin A1	1.8
	12	4.57	316.2599	C_18_H_36_O_4_	Aggreceride A	1.75
	13	1.86	321.1425	C_13_H_23_NO_8_	Salbostatin	1.73
*Streptomyces* sp. UR23 with *Nocardia* sp. UR27 in liquid media (**2.4 ± 1.1**)	14	11.85	491.3616	C_29_H_49_NO_5_	Lipstatin	2.29
	15	4.19	287.1518	C_17_H_21_NO_3_	Trichostatic acid	1.87
	16	4.14	302.1626	C_17_H_22_N_2_O_3_	Trichostatin A	1.77
	17	3.71	346.1522	C_18_H_22_N_2_O_5_	Terreusinol	1.71
	18	3.32	370.2339	C_20_H_34_O_6_	Platenolide II	1.68
*Micromonospora* sp. UR17 on solid media **(**7.8 ± 1.2**)**	19	5.13	598.1115	C_32_H_22_O_12_	Crisamicin A	2.33
	20	3.14	158.0934	C_8_H_14_O_3_	Mycinonic acid I	2.11
	21	2.49	168.0414	C_8_H_8_O_4_	Vanillic acid	1.83
	22	2.13	242.0793	C_12_H_10_N_4_O_2_	Lumichrome	1.66
*Micromonospora* sp. UR17 in liquid media (>100)	23	3.91	352.2236	C_20_H_32_O_5_	Neorustmicin D	2.22
	24	2.55	366.1274	C_13_H_22_N_2_O_10_	Trehazolin	1.95
	25	3.96	366.2106	C_14_H_30_N_4_O_7_	Fortimicin-KK1	1.87
*Micromonospora* sp. UR17 with *Nocardia* sp. UR27 on solid media (**2.7 ± 0.7**)	26	3.54	304.1205	C_19_H_16_N_2_O_2_	Neihumicin	1.77
	27	5.89	645.3873	C_36_H_66_NO_9_	Antascomicin D	1.71
	28	5.23	689.4139	C_38_H_59_NO_10_	Antascomicin C	1.65
*Micromonospora* sp. UR17 with *Nocardia* sp. UR27 in liquid media (**2.5 ± 0.9**)	29	4.43	560.2731	C_29_H_40_N_2_O_9_	Geldanamycin	1.86
	30	2.22	152.0467	C_8_H_8_O_3_	2-Hydroxyphenylacetic acid	1.82
	31	3.31	254.0573	C_15_H_10_O_4_	Daidzein	1.73
	32	2.79	234.0888	C_13_H_14_O_4_	GTRI-02	1.66
*Nocardiopsis* sp. UR17 in solid media (>100)	33	2.29	193.0736	C_10_H_11_NO_3_	Nocazoline A	1.84
	34	3.73	336.1473	C_20_H_20_N_2_O_3_	Nocazine C	1.71
*Nocardiopsis* sp. UR17 on liquid media (>100)	35	4.24	369.2418	C_22_H_31_N_3_O_2_	Pendolmycin	1.64
*Nocardiopsis* sp. UR17 with *Nocardia* sp. UR27 on solid media (>100)	36	2.64	125.0471	C_6_H_7_NO_2_	Methyl pyrrole-2-carboxylate	1.99
	37	3.87	139.0272	C_6_H_5_NO_3_	5-hydroxypicolinic acid	1.85
*Nocardiopsis* sp. UR17 with *Nocardia* sp. UR27 in liquid media (>100)	38	2.41	195.0524	C_9_H_9_NO_4_	Forphenicine	1.76
*Nocardia* sp. UR27 on solid media (>100)	39	2.66	248.1310	C_17_H_16_N_2_	Brasilidine A	2.13
	40	1.72	267.0957	C_10_H_13_N5O_4_	Formycin-A	1.92
	41	2.56	282.1204	C_13_H_18_N_2_O_5_	Corynecin III	1.7
*Nocardia* sp. UR27 in liquid media (>100)	42	3.86	284.0790	C_15_H_12_N_2_O_4_	Deoxynyboquinone	1.98
	43	3.7	366.2039	C_20_H_30_O_6_	Spirocardin A	1.73
	44	2.92	368.2188	C_20_H_32_O_6_	Spirocardin B	1.71

* Antitrypanosomal activity (IC_50_) of each extract expressed in µg/mL ± SD. ** VIP stands for variable importance in projection.

**Table 3:** Dereplication table of the significant and highly correlated putatively active metabolites arranged according to their *p*-values.

NO.	Dereplication	Mass	Molecular Formula	Reported Activity	Probability *p* < 0.01
**3**	Desferrioxamine B	560.3537	C_25_H_48_N_6_O_8_	Siderophore	2.42 × 10^−6^
**2**	Nocardamine	600.3487	C_27_H_48_N_6_O_9_	Siderophore	3.33 × 10^−5^
**16**	Trichostatin A	302.1626	C_17_H_22_N_2_O_3_	Histone deacetylase and tyrosinase inhibitor	1.22 × 10^−5^
**10**	Bafilomycin D	604.397	C_35_H_56_O_8_	Insecticidal activity	5.43 × 10^−4^
**28**	Antascomicin C	689.4139	C_38_H_59_NO_10_	Antimicrobial	2.66 × 10^−4^
**11**	Bafilomycin A1	622.4075	C_35_H_58_O_9_	H^+^-ATPase inhibitor	1.12 × 10^−4^
**14**	Lipstatin	491.3616	C_29_H_49_NO_5_	Pancreatic lipase inhibitor	6.89 × 10^−3^
**26**	Neihumicin	304.1205	C_19_H_16_N_2_O_2_	Cytotoxic	3.11 × 10^−3^

## Supplementary Materials

The following are available online at https://www.mdpi.com/2079-6382/9/9/629/s1, Table S1: Panel of *Trypanosoma brucei* targets used for Autodock-Vina calculations; Table S2: Binding energy scores resulting from the docking of OPLS-DA-derived metabolites against the known *T. brucei* molecular targets. Compounds achieving scores <−8 kcal/mol were categorized as top-scoring hits.
