# Supplementary material for: Exploration of Chemical Diversity and Antitrypanosomal Activity of Some Red Sea-Derived Actinomycetes Using the OSMAC Approach Supported by LC-MS-Based Metabolomics and Molecular Modelling
Source: Antibiotics (Basel). 2020 Sep 22;9(9):629. doi: 10.3390/antibiotics9090629 (PMC7558093; doi:10.3390/antibiotics9090629)
Supplement: Supplementary file 1 [file antibiotics-09-00629-s001.pdf]

Supplemental Materials

Exploration of Chemical Diversity and Antitrypanosomal Activity of Some Red Sea-Derived Actinomycetes Using the OSMAC Approach Supported by LC-MS-Based Metabolomics and Molecular Modeling

Noha M. Gamaleldin [1,2,†], Walid Bakeer [3,†], Ahmed M. Sayed [4], Yara I. Shamikh [5,6], Ahmed O. El-Gendy [3], Hossam M. Hassan [7], Hannes Horn [8], Usama Ramadan Abdelmohsen [9,10,*] and Wael N. Hozzein [11,*]

1 Department of Microbiology, Faculty of Pharmacy, The British University in Egypt (BUE), Cairo 11837, Egypt; noha.gamaleldin@bue.edu.eg
2 Center for Drug Research and Development, Faculty of Pharmacy, The British University in Egypt (BUE), Cairo 11837, Egypt
3 Department of Microbiology, Faculty of Pharmacy, Beni-Suef University, Beni-Suef 62514, Egypt; waleed.ismail@nub.edu.eg (W.B.); walidbakeer@pharm.bsu.edu.eg.
4 Department of Pharmacognosy, Faculty of Pharmacy, Nahda University, Beni-Suef 62513, Egypt; Ahmed.Mohamed.Sayed@nub.edu.eg
5 Department of Microbiology & Immunology, Faculty of Pharmacy, Nahda University, Beni-Suef 62513, Egypt; yara.shamikh@nub.edu.eg
6 Virology Department, Egyptian Center for Research and Regenerative Medicine (ECRRM), 11517, Egypt
7 Department of Pharmacognosy, Faculty of Pharmacy, Beni-Suef University, Beni-Suef 62514, Egypt; hossam.mokhtar@nub.edu.eg
8 Independent Researcher, 69126 Heidelberg, Germany; hannes.horn@ch.tum.de
9 Department of Pharmacognosy, Faculty of Pharmacy, Minia University, Minia 61519, Egypt
10 Department of Pharmacognosy, Faculty of Pharmacy, Deraya University, New Minia 61111, Egypt
11 Botany and Microbiology Department, Faculty of Science, Beni-Suef University, Beni-Suef 62514, Egypt
* Correspondence: usama.ramadan@mu.edu.eg (U.R.A.); Wael.Hozzein@science.bsu.edu.eg (W.N.H.)
† These authors contributed equally to this work.

Table S1. Panel of *Trypanosoma brucei* targets used for Autodock-Vina calculations.

Target Name	PDB code	Grid box (Å)
Trypanothione reductase (TR)	2WPF	X= -22.80, Y= -30.72, Z= 60.90
Triosephosphate isomerase (TIM)	1AG1	X= 46.05, Y= 16.79, Z= -10.89
Farnesyl diphosphate synthase (FDS)	2P1C	X= 69.01, Y= 37.02, Z= -2.02
Rhodesain	6EXQ	X= -8.23, Y= 2.3, Z= 11
Ornithine decarboxylase (OD)	1NJJ	X= 20.23, Y= 2.17, Z= 59.78
Sterol 14-alpha demethylase (AD)	3GW9	X= 22.62, Y=38.17, Z= 31.44
HSP90	3OPD	X= -1.36, Y= 16.61, Z= 12.12
Dihydrofolate reductase (DFR)	3QFX	X= -11.38, Y= 31.95, Z= 9.8
Nucleoside 2-deoxyribosyltransferase (NDT)	2F64	X= -27.17, Y= 1.97, Z= -20.94
Rhodesiense adenosine kinase (RAK)	2XTB	X= 18.39, Y= -28.42, Z= 6.5

Table S2. Binding energy scores (kcal/mol) resulted from the docking of OPLS-DA-derived metabolites against the known *T. brucei* molecular targets. Compounds got scores < -8 kcal/mol were categorized as top-scoring hits.

Targets	(2)	(3)	(10)	(11)	(14)	(16)	(26)	(28)
(TR)	**-8.7**	**-10.6**	-4.4	-4.2	-5.3	-6.8	**-8.9**	-5.6
(TIM)	-5.7	-5.9	-4.2	-5.3	-4.6	-6.5	-7.2	-4.9
(FDS)	**-8.6**	**-9.8**	-5.8	-5.4	-7.4	**-8.8**	**-9.1**	-6.1
Rhodesain	-7.4	-3.2	**-8.7**	**-8.9**	-4.2	-5.6	-6.0	-2.3
(OD)	-6.8	-5.9	-.6.3	-6.0	-6.7	-5.4	-4.9	-6.2
(AD)	-4.8	-5.2	-4.0	-5.3	-6.3	-4.7	-4.9	-5.8
HSP90	-5.5	-5.0	-6.9	-6.2	-5.8	-7.3	-7.4	-6.4
(DFR)	-4.3	-3.4	-4.9	-5.3	-5.7	-6.9	-7.4	-5.9
(NDT)	-4.1	-3.8	-3.5	-4.0	-4.9	-6.9	-7.2	-4.9
(RAK)	-4.0	-3.1	-4.9	-5.3	-4.0	-6.6	-5.3	-5.1
